# Heterogeneity of interactions of microbial communities in regions of Taihu Lake with different nutrient loadings: A network analysis

**DOI:** 10.1038/s41598-018-27172-z

**Published:** 2018-06-11

**Authors:** Xinyi Cao, Dayong Zhao, Huimin Xu, Rui Huang, Jin Zeng, Zhongbo Yu

**Affiliations:** 1 State Key Laboratory of Hydrology-Water Resources and Hydraulic Engineering, College of Hydrology and Water Resources, Hohai University, Nanjing, 210098 China; 2 State Key Laboratory of Lake Science and Environment, Nanjing Institute of Geography and Limnology, Chinese Academy of Sciences, Nanjing, 210008 China

## Abstract

To investigate the differences in the interactions of microbial communities in two regions in Taihu Lake with different nutrient loadings [Meiliang Bay (MLB) and Xukou Bay (XKB)], water samples were collected and both intra- and inter-kingdom microbial community interactions were examined with network analysis. It is demonstrated that all of the bacterioplankton, microeukaryotes and inter-kingdom communities networks in Taihu Lake were non-random. For the networks of bacterioplankton and inter-kingdom community in XKB, higher clustering coefficient and average degree but lower average path length indexes were observed, indicating the nodes in XKB were more clustered and closely connected with plenty edges than those of MLB. The bacterioplankton and inter-kingdom networks were considerably larger and more complex with more module hubs and connectors in XKB compared with those of MLB, whereas the microeukaryotes networks were comparable and had no module hubs or connectors in the two lake zones. The phyla of *Acidobacteria, Cyanobacteria* and *Planctomycetes* maintained greater cooperation with other phyla in XKB, rather than competition. The relationships between microbial communities and environmental factors in MLB were weaker. Compared with the microbial community networks of XKB, less modules in networks of MLB were significantly correlated with total phosphorous and total nitrogen.

## Introduction

Microorganisms are crucial components of aquatic ecosystems, and play important roles in the ecological processes in freshwater lakes^1,2^. Understanding the interactions of microbial communities as well as the relationships between microbial communities and environmental variables in the freshwater ecosystem is a longstanding challenge in microbial community ecology^3^.

In an ecological system, species interact with each other in various ways (such as competition and mutualism), which leads to the formation of complicated networks^3–6^. An understanding of these interactions between taxa in bacterioplankton, microeukaryotes and the inter-kingdom (bacterioplankton and microeukaryotes combined) communities may help us to clarify their functional roles or environmental niches in the ecosystem^7–9^. Co-occurrence is an ecologically important pattern that reflects niche processes that drive coexistence and diversity in biological communities^10–12^. Thus, an analysis of the co-occurrence of microbial systems may help to characterize the biogeography, functional distribution or ecological interactions of microbes.

The composition and diversity of bacterioplankton are closely related to spatial^13,14^, temporal^3,15–17^ and environmental factors, such as temperature, pH and nutrient concentrations^14,18–22^. The composition of a microbial community differs throughout the water column in both natural habitats^23,24^ and manipulated mesocosms^25–27^ under different ecological states or regimes. It has also been observed that the community structure of bacterioplankton changes after nutrients are added to freshwater ecosystems^28^. High nutrient levels have indirect effects through changes in the composition of bacterioplankton, suggesting that the nutritional status of freshwater lakes may be an important factor that determines the structure of the bacterioplankton community^14,24,27^.

Microeukaryotes are an extremely diverse group of organisms with a wide range of distinct morphologies and physiologies, as well as links to higher trophic levels^29–31^. Microeukaryotes differ from bacterioplankton in many regards, such as individual size and use of nutrients^32–34^. Our previous study found that bacterioplankton and microeukaryotes communities differ with respect to composition and assembly processes in regions of Taihu Lake with different nutrient loadings^14^. However, little is known about the relationships between microbial taxa and niches occupied by specific bacterioplankton/microeukaryotes in freshwater ecosystems. The network and co-occurrence patterns of the bacterioplankton and microeukaryotes communities in regions with different nutrient loading levels may be disparate, and it is important to understand how they are influenced by the nutritional status. Furthermore, there is poor understanding of the inter-kingdom interactions based on the method of high-throughput sequencing and network analysis^35^ and it is essential to consider bacterioplankton and microeukaryotes communities together because they are closely functionally associated in organic matter-producing and -recycling processes^35,36^.

In this study, two regions of Lake Taihu [Meiliang Bay (MLB) and Xukou Bay (XKB)] were selected to assess the effect of differences between a high nutrient level and a low nutrient level, respectively, on the interactions among microbial taxa using a network analysis. We sought to answer the following questions: (1) Are the interactions among microbial communities the same in regions with different nutrient loading levels? (2) Are these interactions different between bacterioplankton, microeukaryotes and inter-kingdom? (3) Are the relationships between environmental factors and the network of microbial communities different between regions with different nutrient loading levels?

## Results and Discussion

### Architecture of the networks in the two lake zones

Correlation-based species-species co-occurrence networks were constructed. The degrees of distribution in the four resulting networks all showed a best fit with the truncated power law (Supplemental Fig. S1), indicating the existence of meaningful, non-random associations in networks in the two lake zones. The degrees of distribution also show that, while most of the species were associated with only a few connections, much fewer species had many connections (Supplemental Fig. S1).

**Figure 1:**
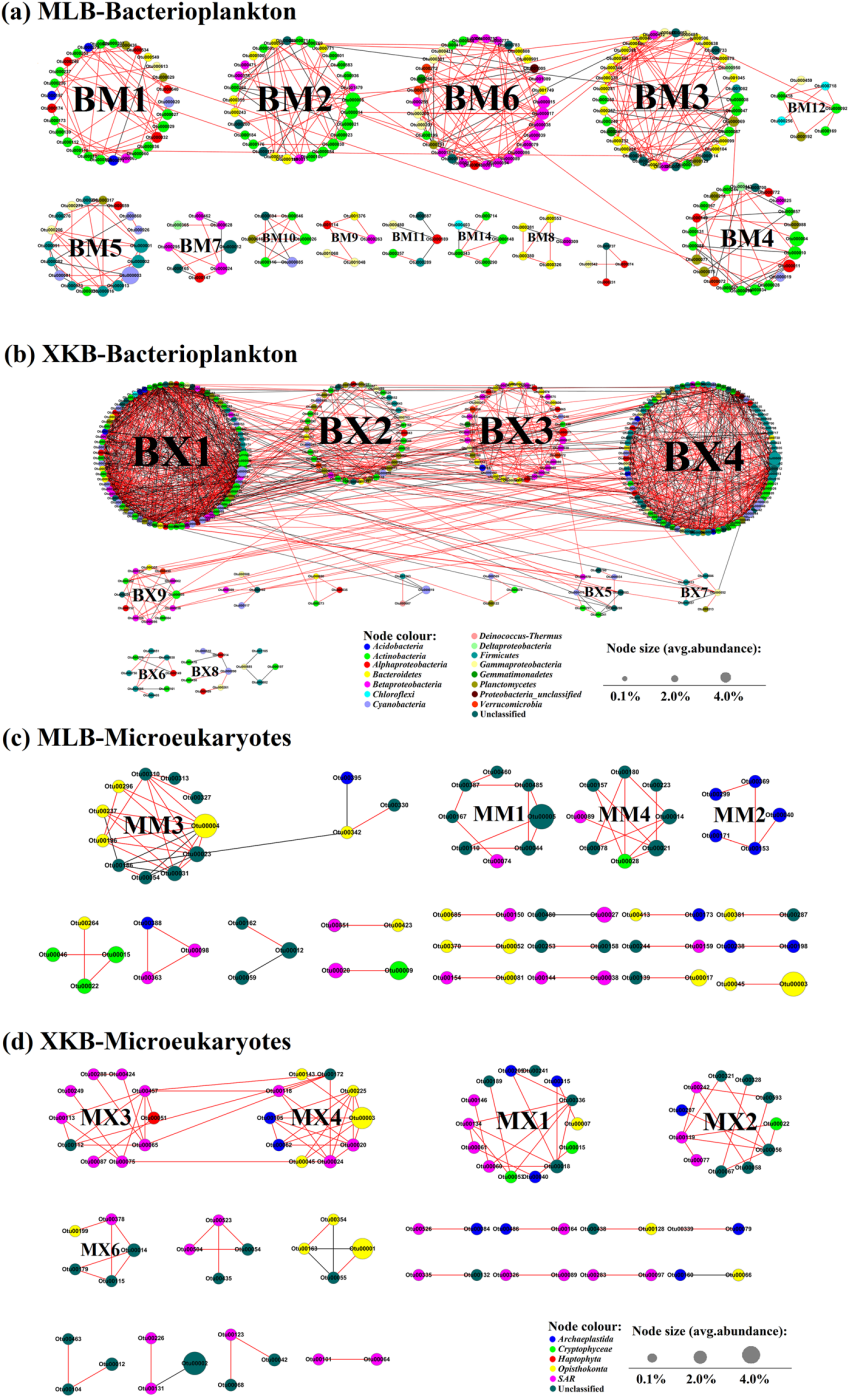
Species-species association network divided by module in MLB and XKB for bacterioplankton and microeukaryotes. Only correlations between species that were statistically significant (*P* < 0.01, Q-value < 0.05) and strong (r ≥ 0.9) were shown. Red solid line means positive correlation and black lines mean negative correlation. Different microbial phyla were represented with different colors and the number on each node means the number of OTUs clustered at 97% similarity. The circles consist of some nodes mean modules. Figure (**a**) and (**b**) represent bacterioplankton networks from MLB and XKB, respectively. Figure (**c**) and (**d**) represent microeukaryotes networks from MLB and XKB, respectively. Modules including less than 4 nodes are removed or abridged for concision.

Comparison of various indexes of the resulting network, including modularity (MD), clustering coefficient (CC), average path length (APL), and network diameter (ND), to those of random networks using Z-tests indicated that the bacterioplankton, microeukaryotes and the inter-kingdom networks in the two regions were non-random (*P* < 0.001) (Table 1). These properties of the observed network were all significantly greater than those of a random network for bacterioplankton and the inter-kingdom network, suggesting that the observed network was more complex than a random network. However, APL and ND of the observed network were significantly lower than those of a random network for microeukaryotes (Table 1). Lower APL and ND of the observed network indicated that the nodes in microeukaryotes network are connected to everyone else through a very short path and facilitate the quick transfer of information more powerful than by chance. The smaller system size (number of nodes and edges) of the microeukaryotes network than bacterioplankton network might lead to this case^37^. For bacterioplankton, microeukaryotes and inter-kingdom communities, the networks in MLB and XKB had higher CC than those in random networks, indicating that there are more highly interconnected (clustered) nodes in the observed networks than in random networks. A small-world network means that most nodes can be reached from every other node by a small number of hops or steps^38^. The clustering nodes in all networks (Fig. 1, Supplemental Fig. S2) and the remarkably high average degree (AD) (Table 1) suggested that these networks have ‘small-world’ properties^38,39^, especially for the microbial community networks in XKB.

**Table 1:** Topological properties of the empirical species-species networks of microbial communities in lake zones with different nutrient loading levels and an associated random network.

Objects	Lake zones	Nodes		Empirical network								Random network			
				Edges		Modularity	Clustering coefficient	Average path length	Network diameter	Average degree	Graph density	Modularity (SD)	Clustering coefficient (SD)	Average path length (SD)	Network diameter (SD)
Bacterioplankton	MLB	329^b^		436^b^		0.848^a,b^	0.405^a,b^	6.554^a,b^	15^b^	2.63^b^	0.008	0.652 (0.010)	0.008 (0.005)	5.737 (0.138)	13.41 (1.261)
	XKB	353^b^		1443^b^		0.546^a,b^	0.472^a,b^	4.674^a,b^	12^a,b^	8.21^b^	0.023	0.314 (0.005)	0.023 (0.003)	3.017 (0.005)	5.263 (0.445)
Microeukaryotes	MLB	73^b^		73^b^		0.819^a^	0.535^a^	2.038^a,b^	5^a,b^	2^b^	0.028	0.658 (0.028)	0.029 (0.028)	5.134 (0.568)	12.229 (2.033)
	XKB	85^b^		98^b^		0.813^a^	0.523^a^	2.556^a,b^	6^a,b^	2.31^b^	0.027	0.626 (0.024)	0.026 (0.021)	4.781 (0.333)	11.217 (1.523)
Inter-kingdom	MLB	Bacterioplankton	287 ^b^	Intra-kingdom	326 (31^c^)^b^	0.899^a,b^	0.327^a,b^	6.540^a,b^	17^a,b^	2.15^b^	0.006	0.735 (0.013)	0.006 (0.006)	6.961 (0.296)	16.880 (1.774)
		Microeukaryotes	63^b^	Inter-kingdom	51^b^										
	XKB	Bacterioplankton	369^b^	Intra-kingdom	1449 (96)^b^	0.579^a,b^	0.432^a,b^	5.227^a,b^	16^a,b^	7.43^b^	0.015	0.335 (0.005)	0.015 (0.002)	3.306 (0.006)	6.049 (0.266)
		Microeukaryotes	116 ^b^	Inter-kingdom	352^b^										

Random networks were generated by rewiring all of the links with the same numbers of nodes and edges to the corresponding empirical network. The numbers in parentheses indicate the standard deviation (SD) of topological properties of 1000 random networks. MLB, lake zone with high nutrient loading; XKB, lake zone with low nutrient loading.

^a^Significant difference (*P* < 0.001) between the empirical network and the random network (Z-test).

^b^Significant difference (*P* < 0.001) between network indexes for the two lake zones (Student t-test).

^c^Numbers in parenthesis represent the number of microeukaryotes-microeukaryotes edge in the inter-kingdom network.

Although identical thresholds were used to define the networks, the sizes of the networks in the two regions were different (Table 1). The network properties for bacterioplankton in MLB were closer to those of random networks (Table 1). Our previous study found that stochastic processes played non-negligible roles in controlling the assembly of both bacterioplankton and microeukaryotes communities in MLB^14^. The higher nutrient loadings in MLB may account for the similarity to random networks in this region.

As shown in Figs 1 and S2, the network in XKB was significantly larger and more complex than that in MLB for bacterioplankton and inter-kingdom, but the networks in the two regions were comparable for microeukaryotes. Significant differences in all of the network indexes were observed between MLB and XKB for the bacterioplankton as well as the inter-kingdom networks (Student’s t-test, *P* < 0.001) (Table 1). However, there were no significant differences in MD or CC (*P* > 0.05) between the microeukaryotes networks in the two regions. The greater complexity of the network in XKB (Fig. 1) may be due to the relatively lower nutrient loading level, which would lead to relatively stronger niche selection due to the greater competition for resources and less-diverse resources^6^. It has been suggested that bacterioplankton and microeukaryotes have similar cellular-mineral-environmental constraints^40^ and it is well known that bacterioplankton and microeukaryotes are very sensitive to environmental variations^14,35^. Although the lower nutrient loading and stronger environmental filtering effects in XKB may lead to stronger niche selection, microeukaryotes are better able to adapt to environmental perturbations and compete with each other^40^, which contributes to the comparable microeukaryotes networks in MLB and XKB as well as the correlations between microeukaryotes and environmental variables (Supplemental Fig. S3).

### Co-occurrence/co-exclusion patterns in the two regions

The MD values of networks for bacterioplankton (MLB: 0.848, XKB: 0.546), microeukaryotes (MLB: 0.819, XKB: 0.813) and inter-kingdom (MLB: 0.899, XKB: 0.579) were all >0.50 (Table 1), and thus had modular structures^17,41,42^. Therefore, the species-species association networks were divided according to modules and obvious differences were found for the networks of bacterioplankton (Fig. 1) and inter-kingdom (Supplemental Fig. S2) in MLB and XKB.

The network for XKB included more interactions (edges) (Table 1), and the APL and ND of the network for bacterioplankton and inter-kingdom were significant lower in XKB than in MLB (Table 1). However, the average degree (AD), which is the most robust measure of network topology along with CC, of the network for bacterioplankton and inter-kingdom were significant higher for the networks in XKB^43^. In general, there were higher CC and AD but lower APL and ND indexes for the network of XKB, which indicated that the nodes in the network of XKB were more clustered and closely connected with plenty edges for each point, thus the network in XKB was more complex than that for MLB^43^. It is also observed that the interaction of inter-kingdom network was markedly stronger in XKB (195 positive correlations and 157 negative correlations) than those of MLB (39 positive correlations and 12 negative correlations) (Supplemental Table S1). However, the species-species association networks for microeukaryotes communities were not as obviously different as those for bacterioplankton between MLB (Fig. 1c) and XKB (Fig. 1d).

Environmental conditions would affect the interactions among microbial communities. It has been demonstrated that species that share similar ecological niches may exhibit competition when resources are scarce, but may show positive interactions under resource-rich conditions^3,44^. In ecological systems, coexistence is supported by niche processes like environmental filtering^45–47^. Species pairs that co-occur may share similar ecological characteristics^8,9,48^, which can be used to infer life-history strategies^49,50^. Glöckner *et al*.^51^ found that bacterioplankton and microeukaryotes showed significant differences in the abundance and relationships among phyla under different nutrient loading conditions. The favorable nutrient state in MLB could weaken niche selection by providing more resources and reducing competition among species, which may explain the weak correlation and simple network^28,52^ in MLB. However, the microeukaryotes networks in the two regions were comparable. It has been demonstrated that, in pelagic ecosystems, microeukaryotes are the primary consumers of phytoplankton, heterotrophic bacteria and archaea^34,53^. In addition, they serve as important trophic links for transferring carbon between the microbial food web and the metazoan food web^34^. Furthermore, uncommonly delineated microeukaryotes species may contribute to the difference between networks for bacterioplankton, inter-kingdom community and microeukaryotes in the two lake zones^14,54^.

### Intra- and inter-phyla co-occurrence/co-exclusion patterns for bacterioplankton

The O/R ratios (O: observed incidence of the co-occurrence of two taxa; R: random incidence of the co-occurrence of two taxa) for bacterioplankton were calculated to determine if OTUs from the same phylum/different phyla tended to exhibit co-occurrence or co-exclusion. Few significant O/R ratios in microeukaryotes networks were observed and the overwhelming majority of significant O/R ratios in inter-kingdom network were the co-occurrence/co-exclusion patterns for bacterioplankton, thus neither of them was shown. An O/R value > 1 means that the observed incidence of co-occurrence (O) of two taxa was higher than that expected at random. As shown in Supplemental Table S2, the O/R values were almost all significantly higher than 1 in both XKB and MLB for positive interaction, indicating a very strong co-occurrence pattern for intra-phylum OTUs, especially in MLB (O/R values almost all > 3). However, the O/R values for negative intra-phylum interaction were almost 0 or NA, and not significant in both XKB and MLB. Co-occurrence reflects commonly preferred conditions or cooperative behaviors^50^; the higher nutrient loading in MLB provided more suitable environmental conditions for these phyla to live and co-occur. It has been demonstrated that strong ecological intra-phyla linkages are due to synergistic relationships, and species from the same phylum tend to co-occur^55^. Phylogenetic signal analysis also revealed that closely related OTUs have similar habitat associations^49,56^, which is consistent with our results.

The O/R values for inter-phyla interactions in XKB were more significant than those in MLB for both positive and negative interactions. For example, *Chloroflexi* showed significant and strong co-occurrence relationships (O/R > 3, *P* < 0.01) with many phyla (*Acidobacteria*, *Actinobacteria*, *Cyanobacteria*, *Gemmatimonadetes*, *Planctomycetes*, *Betaproteobacteria* and unclassified) in the bacterioplankton community of XKB, but showed no significant co-occurrence patterns in MLB. Other phyla, such as *Acidobacteria* (with *Actinobacteria*, *Chloroflexi*, *Cyanobacteria*, *Gemmatimonadetes* and *Planctomycetes*), *Cyanobacteria* (with *Acidobacteria*, *Bacteroidetes*, *Betaproteobacteria*, *Chloroflexi*, *Gammaproteobacteria*, *Gemmatimonadetes*, *Planctomycetes* and unclassified) and *Planctomycetes* (with *Acidobacteria Actinobacteria*, *Bacteroidetes*, *Betaproteobacteria*, *Chloroflexi*, *Cyanobacteria*, *Firmicutes* and *Gemmatimonadetes* and unclassified), showed a similar difference in co-occurrence patterns as *Chloroflexi* in the two lake zones.

The phyla of *Acidobacteria*, *Cyanobacteria* and *Planctomycetes* maintained strong relationships with other phyla in XKB (Supplemental Table S2), which may reflect greater cooperation with other phyla, rather than competition. Therefore, once in a suitable environment, bacterioplankton will live, and intra-phyla co-occurrence will usually occur. On the other hand, an unsuitable environment would dramatically increase inter-phyla relationships. Thus, there were more relationships, such as symbiosis or competition, among species in XKB than in MLB, indicating that the networks between bacterioplankton may change with different nutrient loading levels.

### Detection of the topological roles of nodes for bacterioplankton and inter-kingdom network

**Figure 2:**
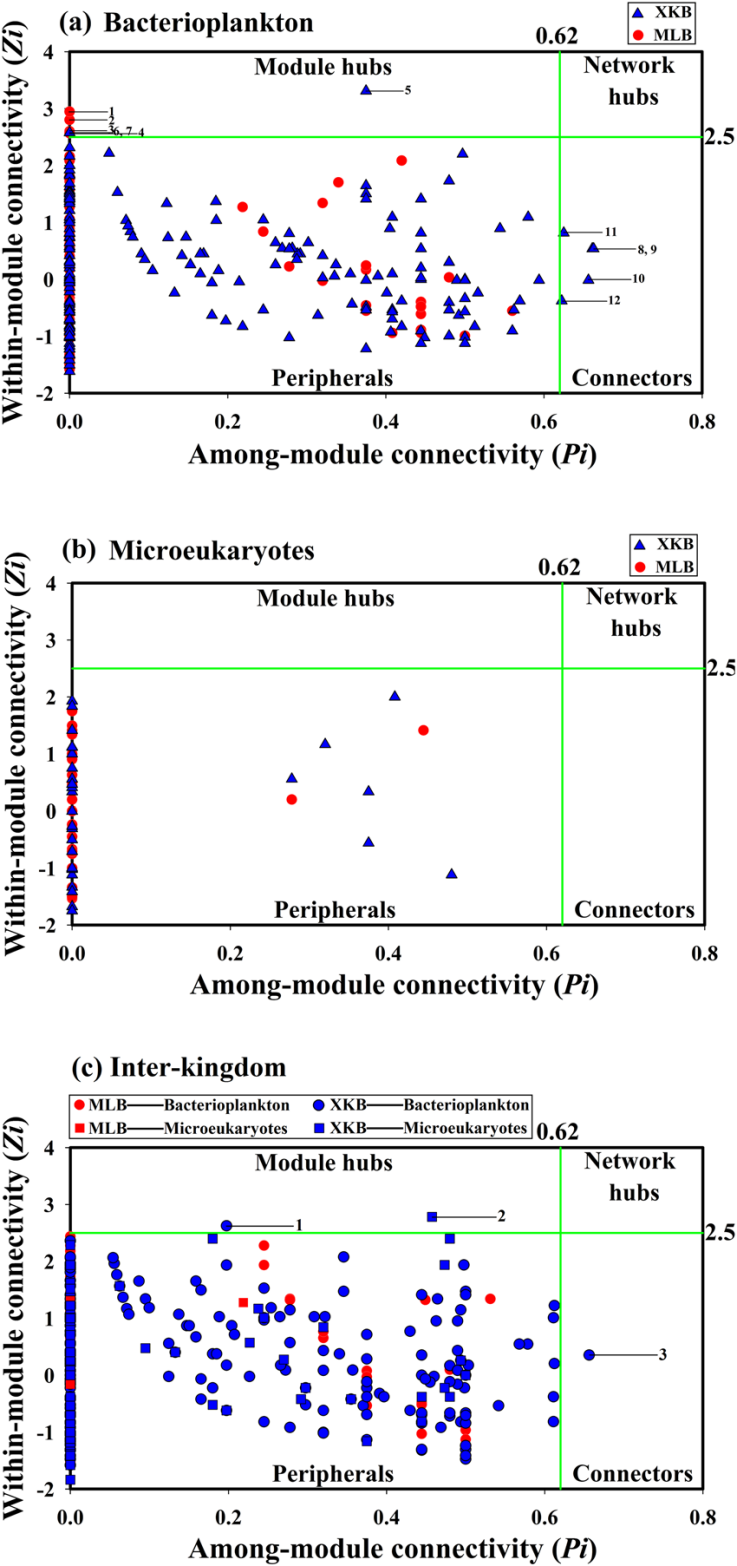
Zi-Pi plot showing the distribution of OTUs based on their topological roles. Each symbol represents an OTU in MLB (red) or XKB (blue) for bacterioplankton (**a**) microeukaryotes (**b**) and inter-kingdom (**c**). The topological role of each OTU was determined according to the scatter plot of within-module connectivity (Zi) and among-module connectivity (Pi). The module hubs and connectors are labeled with numbers.

The topological roles of the OTUs identified in these four networks are shown as a Zi-Pi plot (Fig. 2). The detection of the topological roles for microeukaryotes was omitted because there was neither module hub nor connector in the microeukaryotes network. It was observed that most of the OTUs (98.8% and 97.7% for MLB and XKB, bacterioplankton, respectively; 100% and 99.38% for MLB and XKB, inter-kingdom, respectively) were peripherals, with most of their links inside their modules. Furthermore, among these peripherals, most OTUs (91.7% and 67.8% for MLB and XKB, bacterioplankton, respectively; 92.71% and 82.08% for MLB and XKB, inter-kingdom, respectively) had no links outside their own modules (Pi = 0). For the network of bacterioplankton, 4 (1.2%) OTUs were identified as module hubs network of MLB, 3 (1.4%) and 5 (0.9%) OTUs were identified as module hubs and connectors in the network of XKB respectively (Fig. 2a and Table 2). For the network of inter-kingdom, 2 (0.41%) and 1 (0.21%) OTUs were identified as module hubs and connectors in the network of XKB respectively (Fig. 2c and Table 2), no connectors or module hubs were observed in the network of MLB for inter-kingdom (Fig. 2c and Table 2). It is worth mentioning that OTUE00117 (phylum-*Archaeplastida*) was identified as module hubs in the network of XKB for inter-kingdom (Table 2), showing the important component of microeukaryotes in the whole ecosystem^35,36^. It is reported that the *Archaeplastida* are a major group of microeukaryotes, which may be the reason why it was the module hubs of the network^57^. Furthermore, no network hubs were observed in all of the networks in two lake zones (Fig. 2). Table 2 shows that all of the module hubs were from different modules (BM1, BM2, BM3 and BM6 for bacterioplankton; X3, X4, X12 for inter-kingdom) in the network of MLB for bacterioplankton and both lake zones for inter-kingdom. However, for bacterioplankton in XKB, module hubs were mostly from module BX3 while the connectors were mostly derived from module BX2 and were classified into different phyla (*Planctomycetes*, *Actinobacteria* and unclassified). The other two connectors from modules BX7 and BX4 were classified as *Gammaproteobacteria* and *Cyanobacteria* (Table 2). Furthermore, Table 2 shows that all of the module hubs and connectors were from different phyla in the specified region (*Bacteroidetes*, *Alphaproteobacteria*, *Actinobacteria* and *Gemmatimonadetes* for MLB, *Actinobacteria*, *Alphaproteobacteria* and *Betaproteobacteria* for XKB) and *Alphaproteobacteria* and *Actinobacteria* appeared to be module hubs in both MLB and XKB. Members of the *Actinobacteria* and *Alphaproteobacteria* were widely present in both MLB and XKB (Fig. 1). Hub OTUs in the co-occurrence network mostly belonged to *Actinobacteria* and *Alphaproteobacteria* (Table 2), suggesting that some OTUs of *Actinobacteria* and *Alphaproteobacteria* play important roles in the networks in the two regions. Associations among bacterioplankton are usually established by a cluster of multiple highly interacting species with similar ecological niches or cooperation^28^. *Actinobacteria* play an important role in the decomposition of organic materials and the production of secondary metabolites with very diverse activities^28,58^, which may result in their influential status. It has been reported that *Alphaproteobacteria* isolates can either promote or inhibit the growth of coexisting blooming *Cyanobacteria* in freshwater lakes, which implies a strong functional interaction^3^. In other studies, *Alphaproteobacteria* were prominent members of modules at all time points and co-occurred with *Actinobacteria* and other phyla^59^, which may explain the module-hub role of *Alphaproteobacteria*. Module hubs in the MLB bacterioplankton community network ensured that species within a module were linked more tightly, which explains why the MLB network had much higher modularity than XKB (Table 1). Similarly, connectors in the XKB network made much tighter interactions among modules than those in MLB, which also confirmed that the network structures in the two regions were different.

**Table 2:** Module hubs and connectors in the species-species association networks for bacterioplankton and inter-kingdom community.

Objects	Lake zones	Type of points	Node	OTU ID	Module	Mean abundance (%)	Kingdom	Phylum/Subphylum	Lowest taxonomic rank
Bacterioplankton	MLB	Module Hubs	1	Otu000400	BM3	0.58	Bacterioplankton	Bacteroidetes	c_Bacteroidetes^a^
			2	Otu000032	BM1	2.14	Bacterioplankton	Alphaproteobacteria	f_Rhizobiales
			3	Otu000005	BM2	5.62	Bacterioplankton	Actinobacteria	f_Actinomycetales
			4	Otu000266	BM6	0.69	Bacterioplankton	Gemmatimonadetes	g_Gemmatimonadaceae
	XKB	Module Hubs	5	Otu000110	BX2	0.92	Bacterioplankton	Actinobacteria	f_Actinomycetales
			6	Otu000011	BX3	3.2	Bacterioplankton	Alphaproteobacteria	g_Candidatus_Pelagibacter
			7	Otu000094	BX3	0.93	Bacterioplankton	Betaproteobacteria	g_Comamonadaceae
		Connectors	8	Otu000191	BX2	0.85	Bacterioplankton	Planctomycetes	g_Planctomycetaceae
			9	Otu000173	BX2	0.81	Bacterioplankton	Actinobacteria	g_Acidimicrobiaceae
			10	Otu000361	BX2	0.53	Bacterioplankton	unclassified	p_Bacteria
			11	Otu000052	BX7	1.23	Bacterioplankton	Gammaproteobacteria	g_Xanthomonadaceae
			12	Otu000186	BX4	0.82	Bacterioplankton	Cyanobacteria	g_GpIIa
Inter-kingdom	XKB	Module Hubs	1	OtuB00005^b^	X4	2.64	Bacterioplankton	Actinobacteria	f_Actinomycetales
			2	OtuE00117	X3	0.40	Microeukaryotes	Archaeplastida	g_Chlorophyceae
		Connectors	3	OtuB00167	X12	0.64	Bacterioplankton	Actinobacteria	o_Actinobacteria

^a^p_, c_, o_, f_ and g_ represent phylum, class, order, family and genus, respectively. MLB, Meiliang Bay; XKB, Xukou Bay.

^b^OtuB represents the OTU in bacterioplankton network. OtuE represents the OTU in the microeukaryotes network. Other ID of OTUs are in line with this case.

### Relationships between modules and environmental factors

To explore the effects of environmental factors on species-species association networks, environmental factors were added to these networks (Supplemental Figs S3 and S4, in which red lines signify positive correlations and black lines signify negative correlations). Compared with the environmental variables in MLB, those in XKB had greater effects on these modules (Supplemental Figs S3 and S4). Most nodes in modules BX1, BX2 and BX4 for bacterioplankton (Supplemental Fig. S3) and modules X1 and X2 for inter-kingdom (Supplemental Fig. S4) network were positively correlated with total phosphorous (TP) and total nitrogen (TN) (Supplemental Table S3).

**Figure 3:**
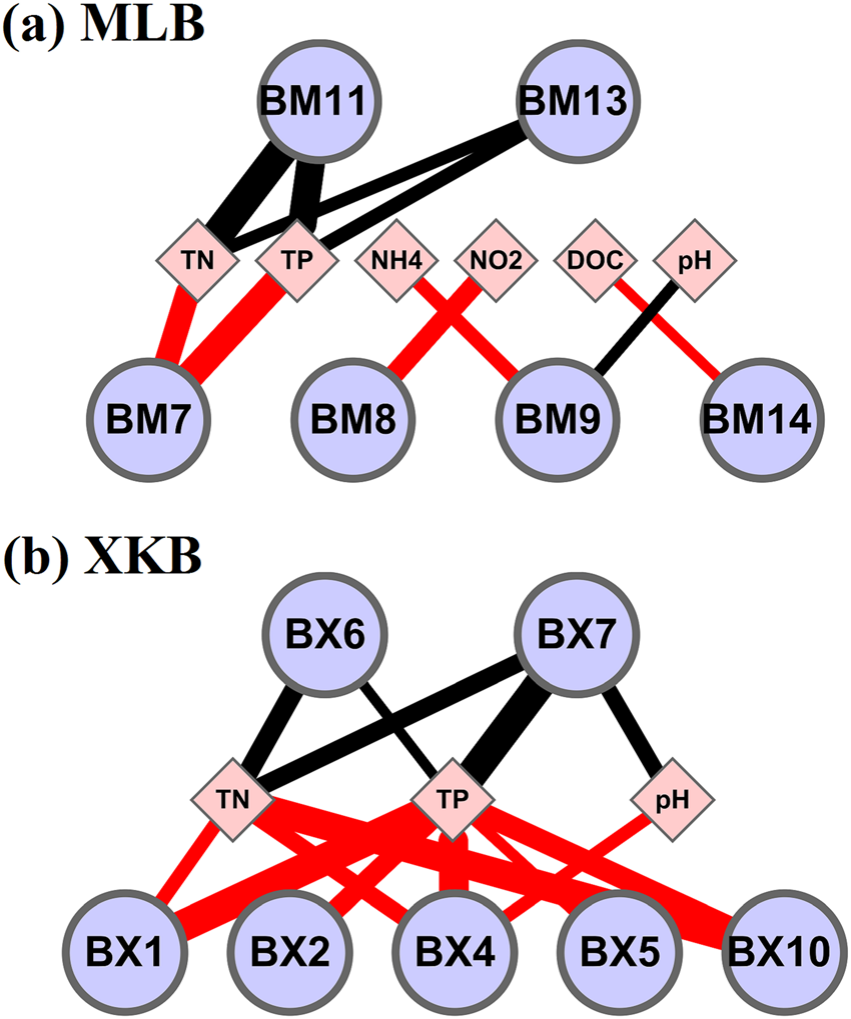
Environmental eigengene networks uncovered relationships between modules (based on the MLB (**a**) and XKB (**b**) network) and environmental variables for bacterioplankton. Only correlations that are statistically significant (*P* < 0.05) are shown. The line thickness is proportional to the absolute value of the Spearman’s correlation coefficient. Node labels stand for environmental variables or the eigengene of a module. The red solid line signifies a positive correlation, and the black line signifies a negative correlation. Environmental variables: TN, total nitrogen; TP, total phosphorus; NH_4_, ammonia nitrogen; NO_2_, nitrite; DOC, dissolved organic carbon.

An eigengene analysis^60^ was performed for modules that were positively or negatively associated with environmental variables to quantitatively describe the relationships between modules and environmental variables. The eigengene network analysis was omitted for microeukaryotes and inter-kingdom since the module of microeukaryotes and inter-kingdom (MLB) had only a very few significant correlations with environmental variables (Supplemental Table S3). The results of the eigengene analysis on the modules of bacterioplankton networks are shown in Supplemental Fig. S5. Figure 3 shows the relationships between eigengenes in the MLB and XKB networks and environmental variables for bacterioplankton, and represents the first evidence that environmental factors have positive or negative effects on particular bacterioplankton modules. In MLB, module BM7 had significant positive correlations with TN and TP, module BM8 had a significant positive correlation with NO_2_, module BM9 had a significant positive correlation with NH_4_^+^-N and a negative correlation with pH, modules BM11 and BM13 had significant negative correlations with TN and TP, and module BM14 had a significant positive correlation with dissolved organic carbon (DOC) (Fig. 3a). In XKB, modules BX1, BX2, BX4, BX5 and BX10 had significant positive correlations with environmental variables including TN, TP and pH, whereas modules BX6 and BX7 had significant negative correlations with these variables (Fig. 3b). The results regarding the correlations between the module eigengenes and environmental variables in the two regions for bacterioplankton and microeukaryotes networks are shown in Supplemental Table S3.

In our previous study, both nutrient variables (TN, TP) and pH significantly affected the compositions of both the bacterioplankton and microeukaryotes communities in XKB (*P* < 0.05), whereas environmental factors were not significantly related to the composition of the microbial communities in MLB, except for a weak correlation between DOC and the microeukaryotes community^14^. Strom^61^ found that the functional traits of microorganisms are products of multiple populations within these communities rather than those of a single population. All of the results of our previous study are fairly consistent with those shown in Fig. 3, and proved that the modules are composed of bacterial clusters with similar ecological niches.

The relationships between OTUs and ecological relatedness are complicated, and depend on individual microbial groups, as well as environmental conditions^28,61,62^. For instance, nutrient loading had a very strong effect on the modules in both MLB and XKB. Meanwhile, environmental factors affected each module differently (Fig. 3). The node composition is substantially different among different modules in the two regions, since they have different nutrient loadings. The different nutrient loadings suggest different extents of environment stress, which would influence the composition and turnover of the microbial communities^6^. Therefore, it is reasonable that the relationships between the microbial communities and environmental factors in MLB are weaker than those in XKB, since niche selection in the former was weakened by reduced competition.

## Conclusions

The results of the present study demonstrated that the network structure and co-occurrence patterns were significantly different between the MLB and XKB regions of Taihu Lake for bacterioplankton, microeukaryotes and inter-kingdom community. The properties of the obtained networks were significantly different from those of random networks, indicating that the assembly of microbial communities in these lake zones was non-random. The region with lower nutrient loading (XKB), and stronger environmental filtering effects, maintained a higher complexity for the whole network and more complex co-occurrence pattern compared with those in MLB for bacterioplankton and inter-kingdom community. It is also observed that the inter-kingdom interactions were stronger in XKB than those of MLB, whereas the networks for the two regions were comparable for microeukaryotes. Non-random co-occurrence of taxonomically related bacterioplankton was also observed, and OTUs from the same phylum tended to co-occur in both lake zones. The relationships between microbial communities and environmental factors in MLB were weaker than those in XKB for bacterioplankton, microeukaryotes and inter-kingdom community, and environmental factors affected each module differently. This study was limited in that it considered only two lake zones; a wider range of study areas will be needed to determine the impacts of environmental factors on the interactions among microbial communities under distinct nutrient conditions.

## Materials and Methods

### Sample collection, Illumina second-generation sequencing and analysis

Ten water samples were collected from two regions of Taihu Lake (Meiliang Bay and Xukou Bay, which represent different nutrient loadings) in October 2015, respectively (Supplemental Table S4). Physicochemical characteristics of the water samples (including the salinity, temperature, oxidation reduction potential (ORP), pH, and conductivity) were measured *in situ* using a calibrated multifunction water-quality sonde (YSI 6600, Yellow Springs, OH, USA). Other environmental factors (including the TN, TP, NH_4_^+^-N, NO_3_^−^-N, and NO_2_^−^-N) were measured in laboratory as described by Zhao *et al*.^14^. The sample used in the present study were the same as our previous study and the methods of DNA extraction, amplification, pyrosequencing and data analysis have been described^14^. The total OTU richness was 3247 and 2059 at 97% similarity cutoff for all the rarefied samples for the 16S rRNA and 18S rRNA, respectively. The raw reads were deposited into the NCBI Sequence Read Archive (SRA) database (Accession Number: SRP090623).

### The computational procedures for network construction

All samples were divided into two groups [Meiliang Bay (MLB) and Xukou Bay (XKB)] representing higher (MLB) and lower (XKB) nutrient loading levels. To improve the network reliability, only OTUs that appeared in at least 8 samples in each group were considered^63^. The relative proportions of sequence numbers were used for the following correlation analysis, since the sequence numbers of individual OTUs significantly varied among the samples^58^. In each group, a correlation matrix was constructed according to the relative abundance of the OTUs in each sample. Both the correlation matrix (R matrix) and the significance matrix (*P* matrix) were calculated using the ‘*Hmsic*’ package in R by calculating all possible pairwise Spearman rank correlations between all OTUs^64^. Only robust (Spearman correlation coefficient ≥0.9 (or ≤−0.9)) and statistically significant (*P* < 0.01) correlations were considered^65^. The correlation approach was justified by the analysis for the sampling effectuated according to Weiss *et al*.^66^ and then improved by Q-value as described below. The possibility of obtaining false results was reduced by calculating a Q-value, which represented the fraction of false positives or negatives if a given pair was identified to be significant (Q-values < 0.05), using the ‘*qvalue*’ package in R^7^. The node degree (i.e., number of edges connected to the node) was plotted against the probability P(k) that a node would have that degree in the network. Three methods (Power law, Exponential law and Truncated law) of power law-fitting of the degree distribution in networks in the two regions were applied^65^. The existence of meaningful, non-random associations in the networks of the two regions was demonstrated by the structural similarity among these ecological networks, in comparison to a Gaussian connectivity distribution predicted by an expectation of randomness.

### Network characterization

The resulting correlation matrix was transformed into a Cytoscape dataset in R. Cytoscape v2.8.2 was then used^67^ for network visualization and topological analysis. Other information regarding nodes (OTUs), taxonomy, module, edge, weights, and positive and negative correlations, was also imported into Cytoscape.

Each network was separated into modules using the fast greedy modularity optimization^68^. Various indexes, including modularity (MD), clustering coefficient (CC), average path length (APL), network diameter (ND), average degree (AD) and graph density (GD), were used to describe the properties and the overall topologies or structures of the networks. Most of these parameters were calculated using the ‘*igraph*’ packages in R^69^. Random networks were also generated using the ‘*igraph*’ packages in R. For each network in this study, 1000 random networks were generated, and all of the network indexes were calculated individually. The average and standard deviation for each index of all of the random networks were then obtained. The statistical Z-test was used to test the significance of differences between the indexes of the observed and random networks. Student’s t test was used to test the significance of differences between the indexes of the networks in MLB and XKB for bacterioplankton, microeukaryotes and inter-kingdom communities.

### Patterns of co-occurrence and co-exclusion

An R script was developed to check the observed (O) and random (R) incidences of the microbial patterns of co-occurrence and co-exclusion. The O/R ratio has been used as a benchmark for checking non-random assembly patterns in complex bacterial communities^17^. Here, we calculated the observed incidence of the co-occurrence (O) of two taxa as the relative percentage of the number of observed edges between them, whereas the random incidence of co-occurrence (R) was calculated as the mean value of the observed incidence of co-occurrence for 1000 random networks. Hence, the degree of disagreement between the O and R incidences of co-occurrence may be used as a benchmark for exploring non-random assembly patterns in complex microbial communities^51^. In this study, the observed (O) and mean value of the random (R) incidences of co-occurrence and their significance levels were calculated according to Zhao *et al*.^3^. The R code for calculating the O/R has been attached in the supplementary information (Supplemental R code 1).

### Topological roles of individual nodes

Visualization of the topological roles of individual nodes reveals the effects of the nutrient loading level on key microbial populations. Topologically, different OTUs (nodes) play distinct roles in the network^70^. The topological roles of different OTUs can be described by two parameters: within-module connectivity (Zi), which describes how well a node is connected to other nodes within its own module, and connectivity among modules (Pi), which reflects how well a node connects to different modules^69,71^. Zi and Pi are calculated as described by Guimera and Amaral^71^. The R code for calculating the Pi and Zi has been attached in the supplementary information (Supplemental R code 2). According to the simplified classification used in networks^72^, the nodes in a network are divided into four subcategories: (i) peripheral nodes (Zi ≤ 2.5, Pi ≤ 0.62), which have low Z and P values (i.e., they have only a few links and almost always to species within their modules); (ii) connectors (Zi ≤ 2.5, Pi > 0.62), which have a low Z but a high P value (i.e., these nodes are highly linked to several modules); (iii) module hubs (Zi > 2.5, Pi ≤ 0.62), which have a high Z but a low P value (i.e., they are highly connected to many species in their own modules); and (iv) network hubs (Zi > 2.5, Pi > 0.62), which have high Z and P values (i.e., they act as both module hubs and connectors)^58,71^.

### Relationships between modules and environmental variables in the networks of bacterioplankton and microeukaryotes in two lake zones

To investigate the relationships between the distribution of nodes in networks and environmental variables, environmental variables were integrated into the networks. Only correlations between environmental variables and species that were statistically significant (*P* < 0.05) and strong (r ≥ 0.6 or r ≤ −0.6) were considered. In addition, to quantitatively describe the relationships between modules and environmental variables, an eigengene network analysis was performed. In this approach, each module was decomposed into a single representative abundance profile called the module eigengene. The molecular ecological network consisted of many nodes and edges. It was difficult to retrieve information intuitively, but the network could be simplified using various methods, such as module partitioning. Modules can be treated as single units for biologically motivated data reduction^73^. First, all of the nodes in module i were selected, and their eigengene values were calculated using the ‘*WGCNA*’ packages in R^74^. Second, the Spearman correlations were calculated between each eigengene and the environmental variables^3^. The calculations were performed as described by Zhao *et al*.^3^.

## Electronic supplementary material

Supplementary materials
