# Supplementary material for: Heterogeneity of interactions of microbial communities in regions of Taihu Lake with different nutrient loadings: A network analysis
Source: Sci Rep. 2018 Jun 11;8:8890. doi: 10.1038/s41598-018-27172-z (PMC5995825; doi:10.1038/s41598-018-27172-z)
Supplement: Supplementary file 1 — Supplementary materials [file 41598_2018_27172_MOESM1_ESM.doc]

**Heterogeneity of interactions of microbial communities in regions of Taihu Lake with different nutrient loadings: A network analysis**

**(Supplementary Information)**

Xinyi Cao^1,2^, Dayong Zhao^1*^ , Huimin Xu^1,2^, Rui Huang^1,2^, Jin Zeng^2*^, Zhongbo Yu^1^

^1^*State Key Laboratory of Hydrology-Water Resources and Hydraulic Engineering, College of Hydrology and Water Resources, Hohai University, Nanjing, 210098, China;*

^2^*State Key Laboratory of Lake Science and Environment, Nanjing Institute of Geography and Limnology, Chinese Academy of Sciences, Nanjing 210008, China*

**The first two authors contributed equally to this work.**

**Corresponding author**

Name: Dayong Zhao

E-mail address: dyzhao@hhu.edu.cn

Fax number: +86 25 83787891

Telephone number: +86 25 83787891

Name: Jin Zeng

E-mail address: jzeng@niglas.ac.cn

Fax number: +86 25 86882240

Telephone number: +86 25 86882240


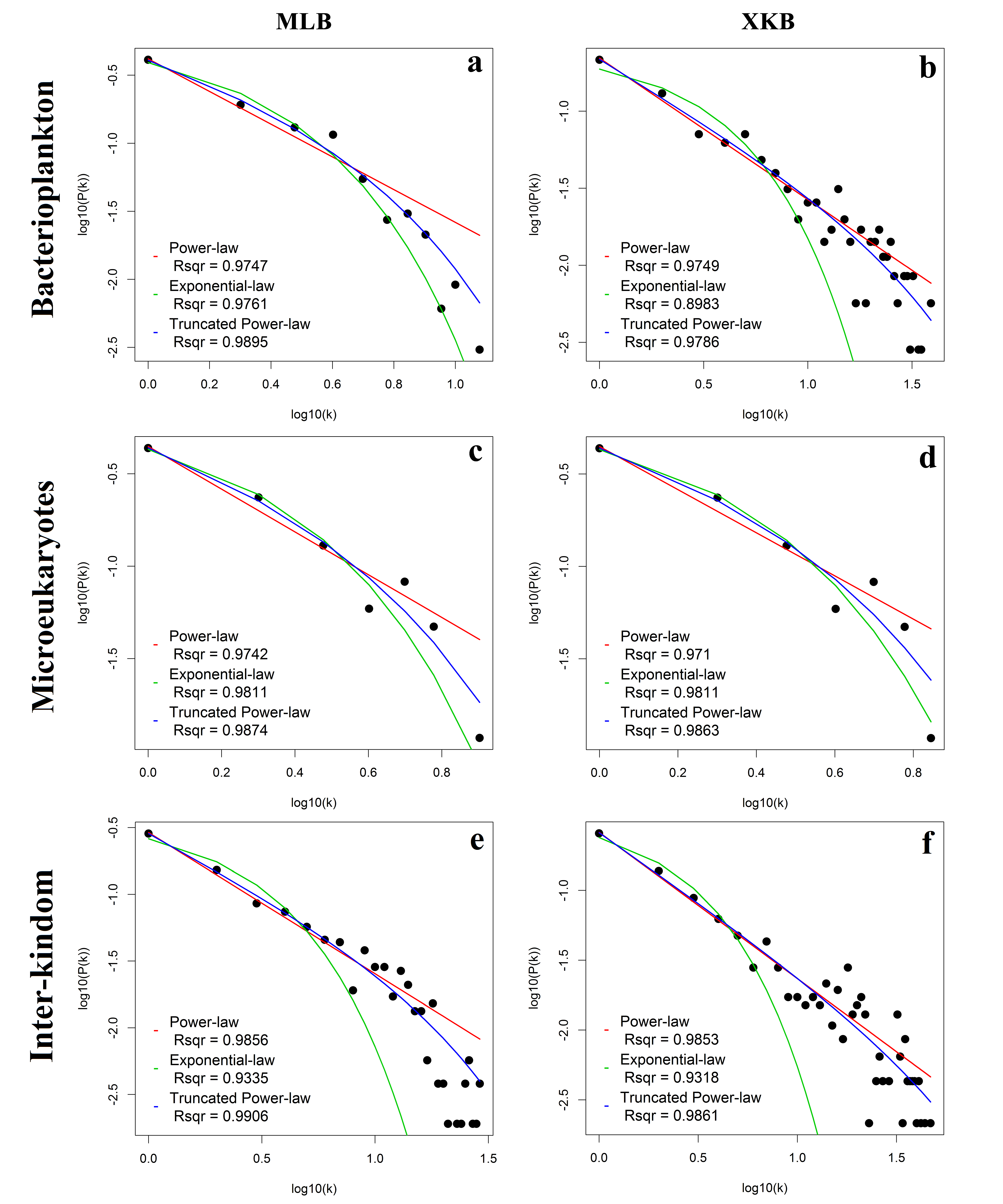


**Figure S1** Degree distribution of the nodes for the networks of bacterioplankton (a, b), microeukaryotes (c, d) and inter-kingdom community (e, f) in MLB and XKB, respectively. The node degree (i.e., the number of edges connected to the node) is plotted against the probability P(k) that a node would have that degree in the network. The red, green and blue line shows the three methods (Power law, Exponential law and Truncated law) of power law fitting of degree distribution in the two lake zones network. For the network, the node degree distribution best follows the Truncated power law distribution, resulting from the preferential attachment of new vertices to the more highly connected vertices. This is quite different from the Poisson shape of the random network of an identical size. This structural similarity among these ecological networks, in contrast with the Gaussian connectivity distribution predicted by the expectation of randomness, also indicated the existence of meaningful, nonrandom associations in the networks of two lake zones.


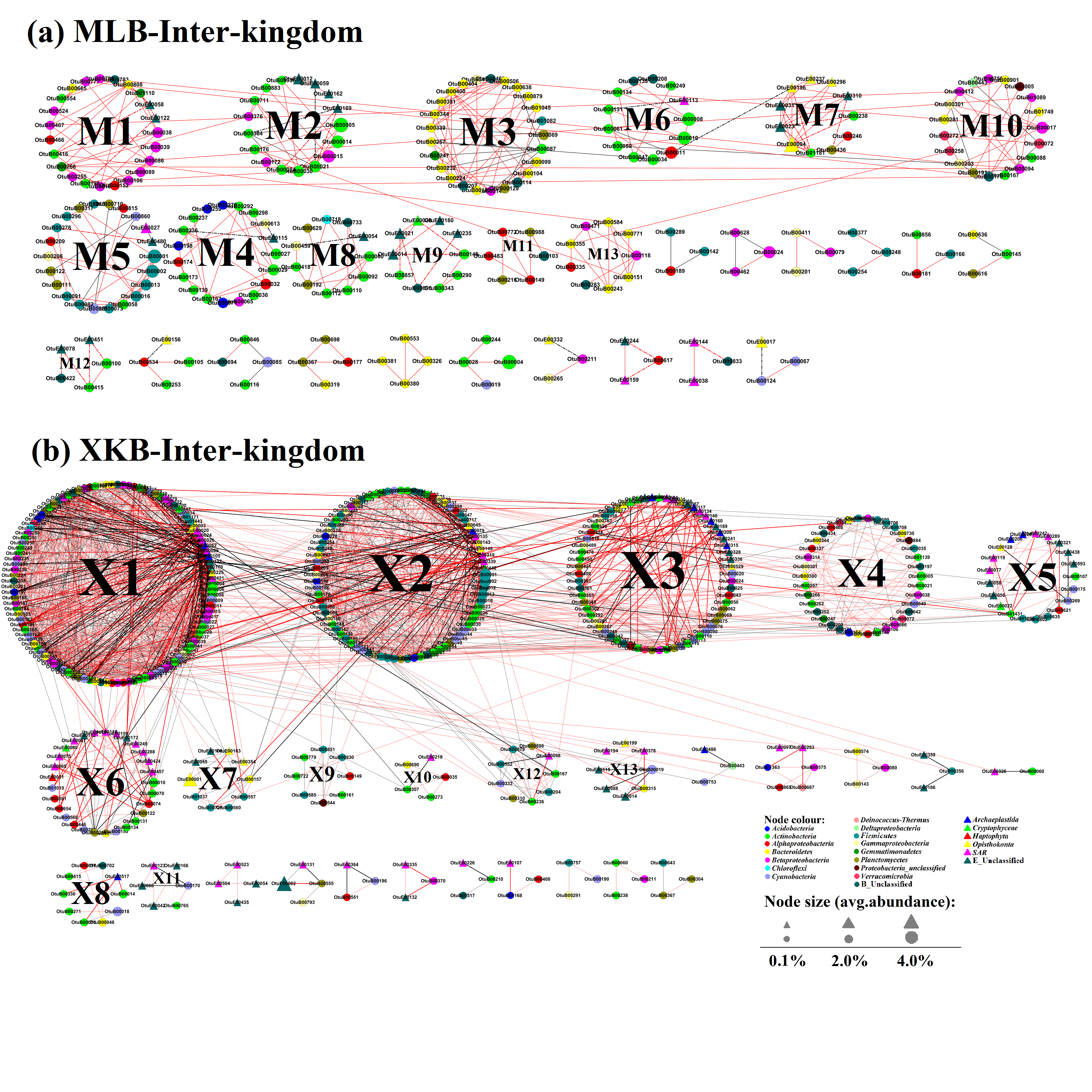


**Figure S2** Species-species association network divided by module and species-environment association networks in MLB (a) and XKB (b) for inter-kingdom community. Only correlations between species that were statistically significant (*P* < 0.01, Q-value < 0.05) and strong (r ≥ 0.9) were shown. Equal-dash red line means positive correlation and black line means negative correlation of the intra-kingdom community. Bold solid red line means positive correlation and black line means negative correlation of inter-kingdom. Different bacterial and microeukaryotic phyla were represented with different colors and shape (ellipse for bacterioplankton, triangle for microeukaryotes), respectively. The number on each node means the number of OTUs clustered at 97% similarity. The circles consist of nodes mean modules. Modules including less than 3 nodes are removed or abridged for concision.


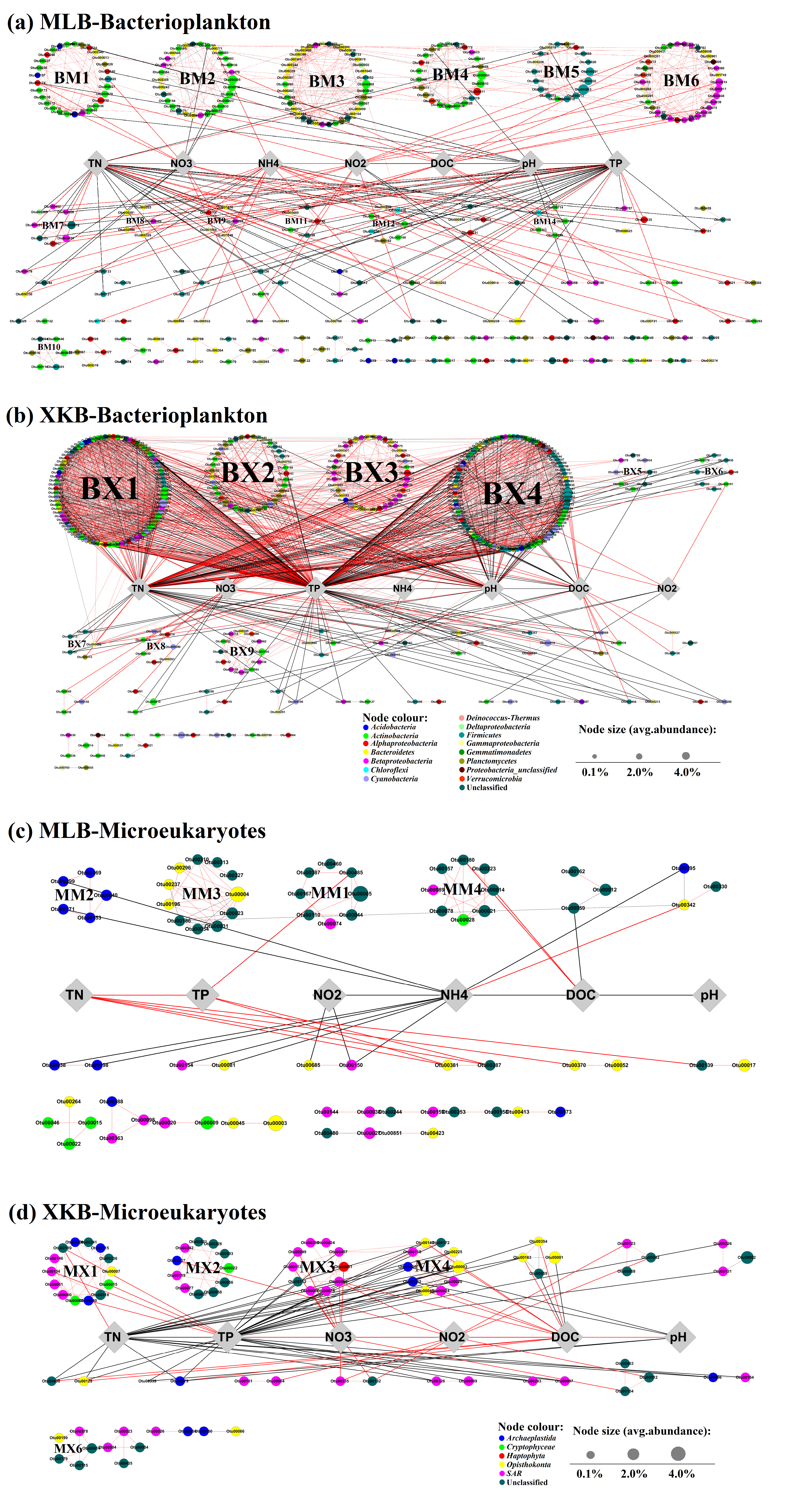


**Figure S3** Species-species association network divided by module and species-environment association network in MLB (a) and XKB (b) for bacterioplankton and MLB (c) and XKB (d) for microeukaryotes. Only correlations between species that were statistically significant (*P* < 0.01, Q-value < 0.05) and strong (r ≥ 0.9) were shown. Only correlations between environmental variables and species that were significant (*P* < 0.05, Q-value < 0.05) were shown. Solid red line means positive correlation and black line means negative species-species correlations . Bold equal-dash red line means positive correlation and black line means negative species-environment correlation. Different bacterial phyla were represented with different colors and the number on each node means the number of OTUs clustered at 97% similarity. The circles consist of some nodes mean modules. Environmental variables, TN, total nitrogen; TP, total phosphorus; NO_3_, nitrate nitrogen; NH_4_, ammonia nitrogen; NO_2_, nitrite; T, temperature, DOC, dissolved organic carbon.


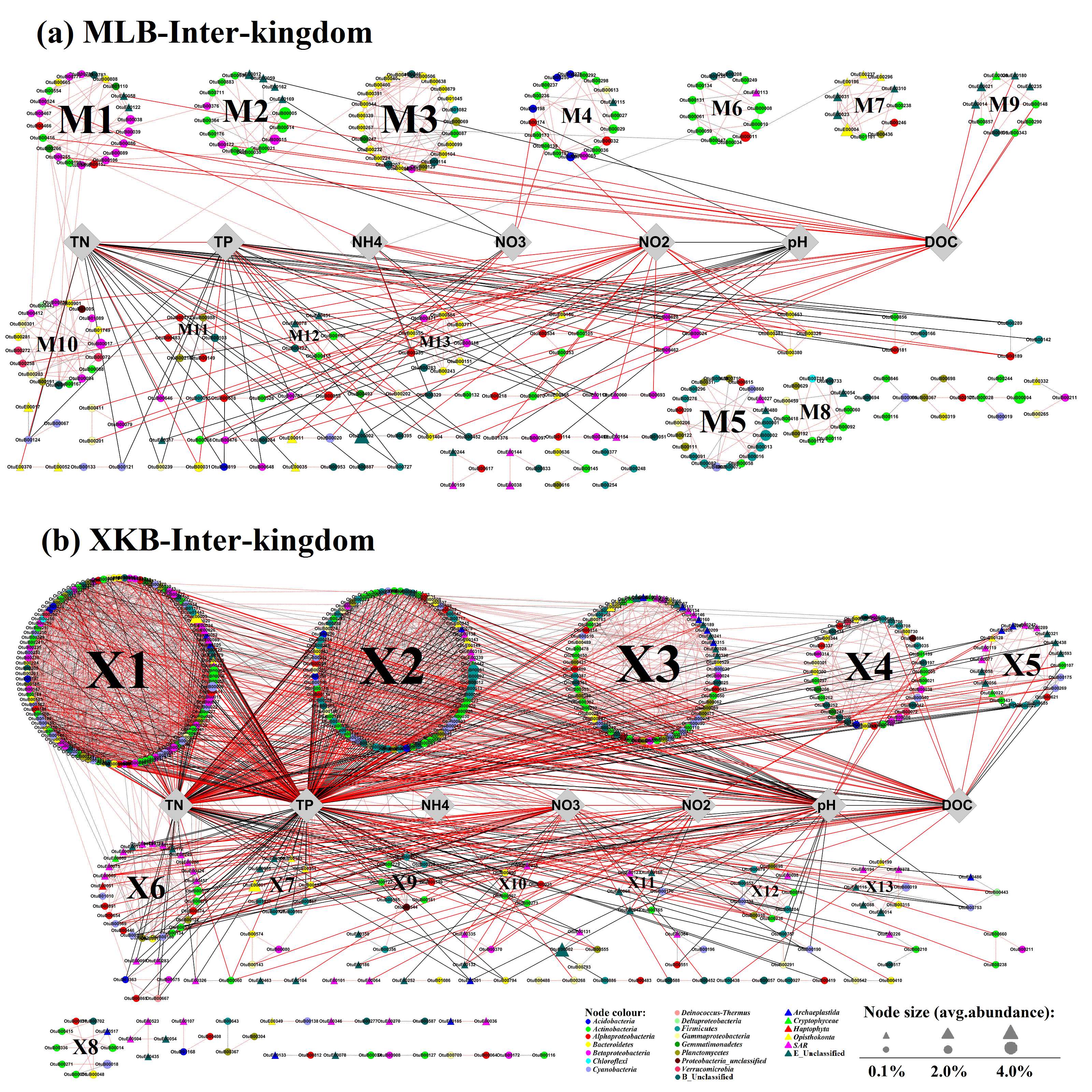


**Figure S4** Species-species association network divided by module and species-environment association network in MLB (a) and XKB (b) for inter-kingdom. Only correlations between species that were statistically significant (*P* < 0.01, Q-value < 0.05) and strong (r ≥ 0.9) were shown. Only correlations between environmental variables and species that were significant (*P* < 0.05, Q-value < 0.05) were shown. Solid red line means positive correlation and black line means negative species-species correlation. Bold equal-dash red line means positive correlation and black line means negative species-environment correlation. Different bacterial and microeukaryotic phyla were represented with different colors and shape (ellipse for bacterioplankton, triangle for microeukaryotes), respectively. The number on each node means the number of OTUs clustered at 97% similarity. The circles consist of some nodes mean modules. Environmental variables, TN, total nitrogen; TP, total phosphorus; NO_3_, nitrate nitrogen; NH_4_, ammonia nitrogen; NO_2_, nitrite; T, temperature, DOC, dissolved organic carbon.


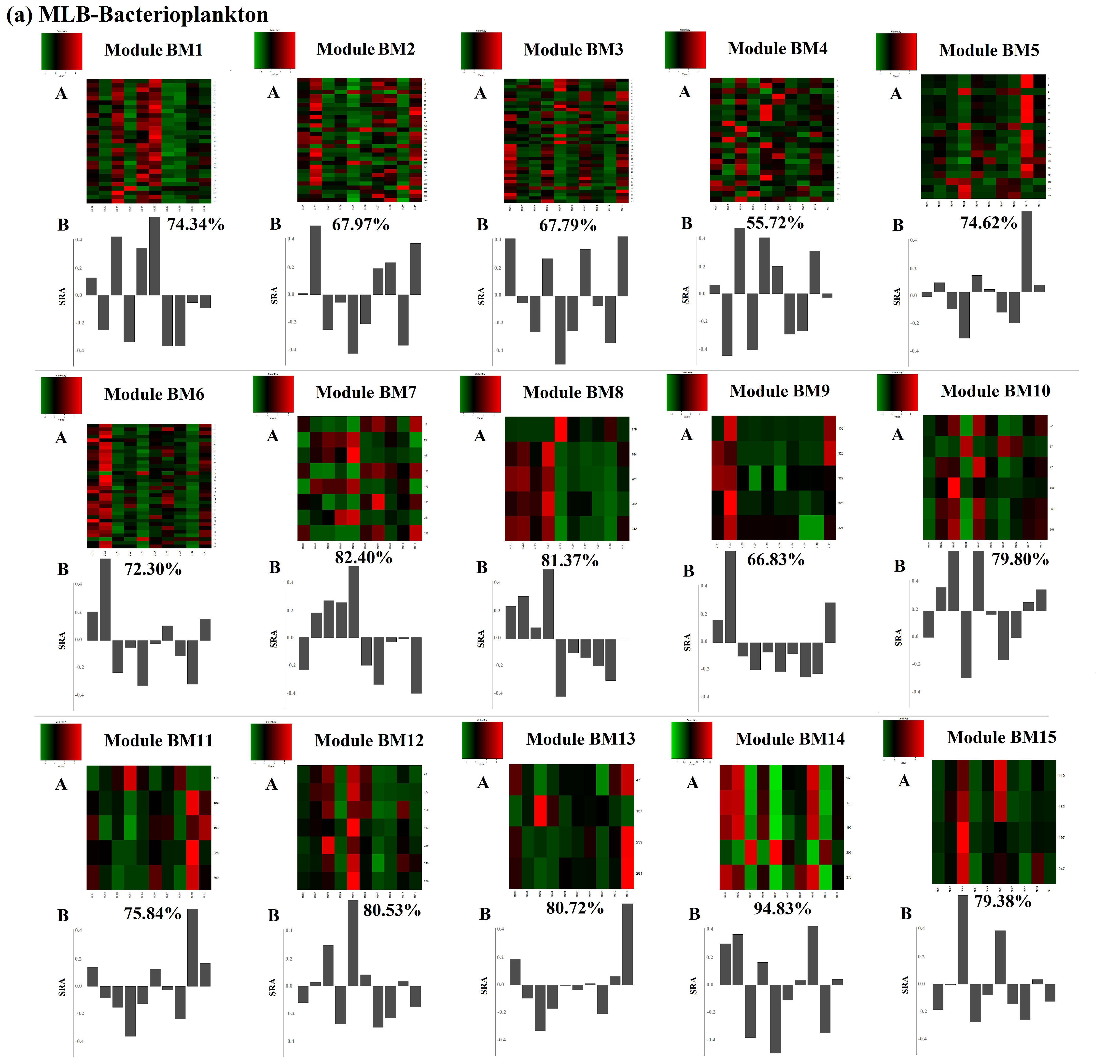


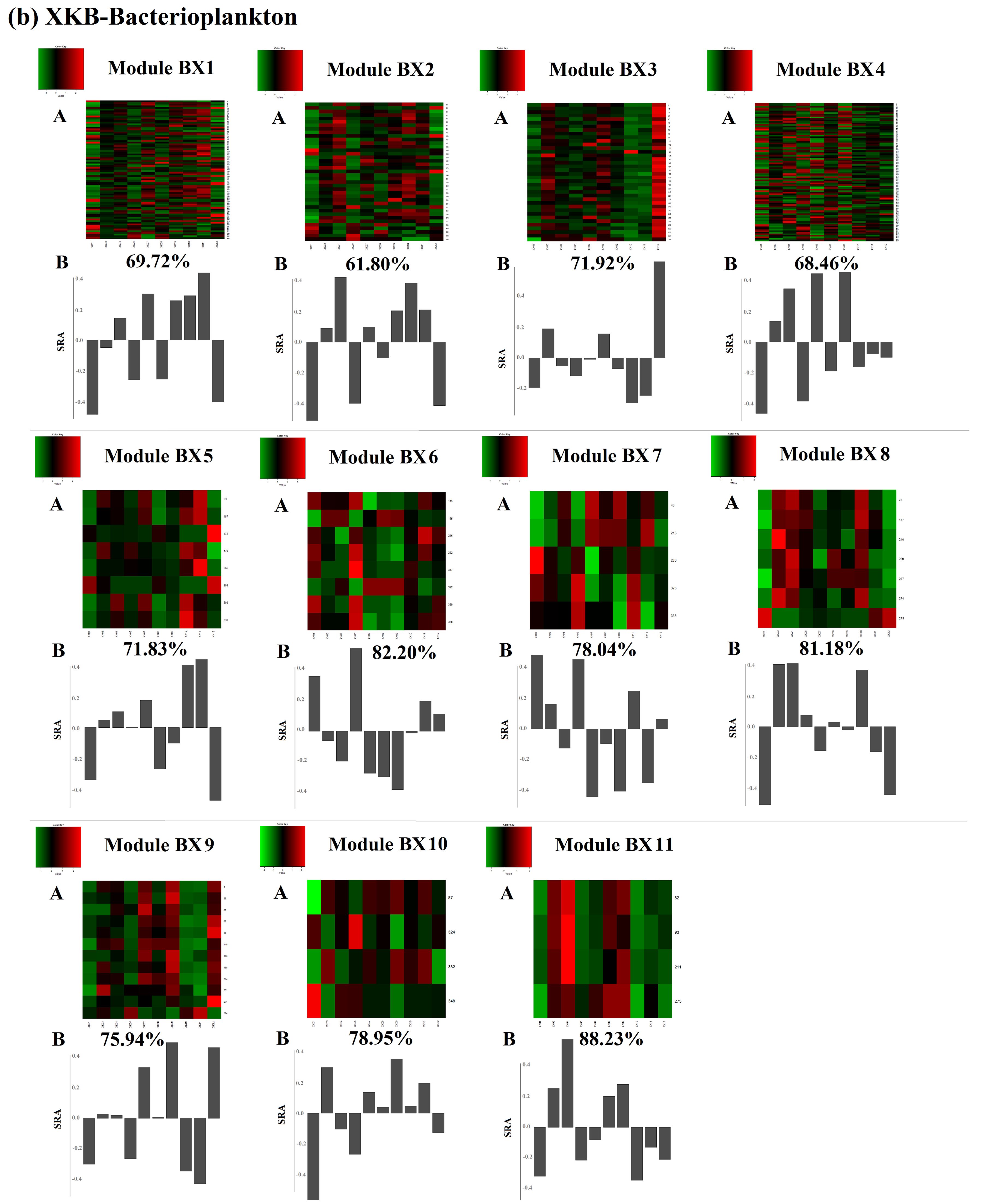


**Figure S5** Eigengene network analysis on the modules of the two lake zones network of bacterioplankton in MLB (a) and XKB (b). For each Figure, (A) Heatmap of the standardized relative abundance (SRA) of bacteria across different samples (rows correspond to OTUs, columns correspond to samples). (B) Expression levels (y-axis) of the corresponding eigengene across the samples (x-axis). The parameter indicates the percentage of the total variance explained by the eigengene.

**Table S1** Number of the positive and negative edges for bacterioplankton, microeukaryotes and inter-kingdom networks in two lake zones.

| Objects | Lake zones | Type of correlation | | Number |
| --- | --- | --- | --- | --- |
| Bacterioplankton | MLB | positive | | 362 |
|  |  | negative | | 74 |
|  | XKB | positive | | 912 |
|  |  | negative | | 531 |
| Microeukaryotes | MLB | positive | | 63 |
|  |  | negative | | 10 |
|  | XKB | positive | | 93 |
|  |  | negative | | 5 |
| Inter-kingdom | MLB | Bacterioplankton-Bacterioplankton | positive | 254 |
|  |  |  | negative | 41 |
|  |  | Microeukaryotes-Microeukaryotes | positive | 29 |
|  |  |  | negative | 2 |
|  |  | Inter-kingdom | positive | 39 |
|  |  |  | negative | 12 |
|  | XKB | Bacterioplankton-Bacterioplankton | positive | 850 |
|  |  |  | negative | 503 |
|  |  | Microeukaryotes-Microeukaryotes | positive | 90 |
|  |  |  | negative | 6 |
|  |  | Inter-kingdom | positive | 195 |
|  |  |  | negative | 157 |

**Table** **S2** Incidence of signiﬁcant and strong (*P* < 0.01, Q-value < 0.05; r ≥ 0.9 or r ≤ -0.9) intra-taxon and inter-taxon co-occurrence/co-exclusion patterns between OTUs. The observed co-occurring/co-exclusion incidence (O) of two taxa was calculated as the relative percentage of the number of observed edges between them in the total edges of the network, whereas the random mean co-occurring/co-exclusion incidence (R) was theoretically calculated by constructing 1000 random networks. The interactions between the OTUs were divided to the positive and negative correlation. When the number of random edges was 0, O/R value was represented by NA. The numbers in bold indicates signiﬁcant differences between the observed incidence and the random mean incidence. *** *P*< 0.001; ** *P*< 0.01; * *P*< 0.05.

| Interaction | Node1 | Node2 | MLB | | XKB | |
| --- | --- | --- | --- | --- | --- | --- |
|  |  |  | positive | negative | positive | negative |
| Intra-taxon Co-occurrence/ Co-exclusion | Acidobacteria | Acidobacteria | **15.38**** | NA | **10.53***** | 0 |
|  | Actinobacteria | Actinobacteria | **2.71***** | 1.39 | **1.63***** | 0.94 |
|  | Alphaproteobacteria | Alphaproteobacteria | 1.86 | 0 | **0.32*** | 0.39 |
|  | Bacteroidetes | Bacteroidetes | **6.09***** | 0 | 1.2 | 0 |
|  | Betaproteobacteria | Betaproteobacteria | **3.45***** | 2.33 | **4.31***** | 1.18 |
|  | Chloroflexi | Chloroflexi | **125**** | 0 | NA | NA |
|  | Cyanobacteria | Cyanobacteria | **7.33***** | 0 | **1.94**** | 1.07 |
|  | Firmicutes | Firmicutes | **10.22***** | 0 | **3.48***** | 0 |
|  | Gammaproteobacteria | Gammaproteobacteria | 1.26 | 0 | 0.35 | 0 |
|  | Planctomycetes | Planctomycetes | 1.09 | 0 | **3.21***** | 0.4 |
|  | Proteobacteria_unclassified | Proteobacteria_unclassified | NA | 0 | 0 | NA |
|  | unclassified | unclassified | 1.25 | 0 | **2.15*** | 0 |
|  | Verrucomicrobia | Verrucomicrobia | **43.48*** | 0 | 0 | 0 |
| Inter-taxon Co-occurrence/ Co-exclusion | Acidobacteria | Actinobacteria | 1.14 | 0 | **3.41***** | 0.81 |
|  | Actinobacteria | Bacteroidetes | **0.28***** | 1.49 | **0.68*** | 0.8 |
|  | Actinobacteria | Betaproteobacteria | **0.47**** | **0.41*** | 0.92 | 1.3 |
|  | Bacteroidetes | Betaproteobacteria | 1.11 | 0.57 | **2.50***** | **0.29*** |
|  | Acidobacteria | Chloroflexi | 0 | 0 | **13.98**** | 0 |
|  | Actinobacteria | Chloroflexi | 1.7 | 2.55 | **6.18***** | 0.79 |
|  | Betaproteobacteria | Chloroflexi | 0 | 0 | 0 | **15.43***** |
|  | Acidobacteria | Cyanobacteria | 0 | 0 | **2.82**** | 1.28 |
|  | Actinobacteria | Cyanobacteria | **0.39*** | 1.25 | **1.63***** | 1.21 |
|  | Bacteroidetes | Cyanobacteria | 0 | 0 | **0.29**** | 1.09 |
|  | Betaproteobacteria | Cyanobacteria | 0 | 0 | **0.47**** | **2.86***** |
|  | Chloroflexi | Cyanobacteria | 0 | 0 | **7.20**** | 3.39 |
|  | Actinobacteria | Firmicutes | **0.16***** | 0 | **0.26***** | **1.24*** |
|  | Alphaproteobacteria | Firmicutes | 0.56 | 2.26 | **0.16***** | 0.65 |
|  | Bacteroidetes | Firmicutes | **0.13**** | 0 | 0 | 0.92 |
|  | Betaproteobacteria | Firmicutes | **0.14**** | 0 | **0.07***** | 0 |
|  | Cyanobacteria | Firmicutes | 0.43 | **26.32***** | **0.37***** | 1.13 |
|  | Actinobacteria | Gammaproteobacteria | **0.17***** | 0 | **0.37***** | 1.02 |
|  | Bacteroidetes | Gammaproteobacteria | 0.69 | 0.88 | **0.46*** | 0.59 |
|  | Cyanobacteria | Gammaproteobacteria | 0 | 0 | **0.47*** | 0.57 |
|  | Firmicutes | Gammaproteobacteria | 1.66 | 0 | **1.48*** | 0.56 |
|  | Acidobacteria | Gemmatimonadetes | 0 | 0 | 4.26 | **15.75**** |
|  | Actinobacteria | Gemmatimonadetes | 0.43 | 1.23 | 0.78 | **4.22**** |
|  | Chloroflexi | Gemmatimonadetes | 0 | 0 | 0 | **37.04*** |
|  | Cyanobacteria | Gemmatimonadetes | 0 | 0 | 0 | **4.46*** |
|  | Acidobacteria | Planctomycetes | 0 | 0 | **2.71*** | 1.92 |
|  | Actinobacteria | Planctomycetes | 0.9 | 1.02 | **1.71***** | 1.09 |
|  | Bacteroidetes | Planctomycetes | **0.13***** | 3.11 | **0.40*** | 1.47 |
|  | Betaproteobacteria | Planctomycetes | 0 | **3.29*** | **0.27***** | **2.91***** |
|  | Chloroflexi | Planctomycetes | 0 | 0 | **13.19***** | 0 |
|  | Cyanobacteria | Planctomycetes | 0.44 | 2.43 | **1.76**** | 1.35 |
|  | Firmicutes | Planctomycetes | 1.58 | 0 | **0.42**** | **0.50*** |
|  | Gemmatimonadetes | Planctomycetes | 0 | 4.5 | 0 | **8.75**** |
|  | Gammaproteobacteria | Proteobacteria_unclassified | 0 | **9.09*** | 0 | 0 |
|  | Actinobacteria | unclassified | 0.85 | 1.13 | **2.08***** | **0.48**** |
|  | Alphaproteobacteria | unclassified | **0.21**** | 2.11 | **0.10***** | **0.19**** |
|  | Betaproteobacteria | unclassified | 1.36 | 0.67 | **0.44*** | 1.78 |
|  | Chloroflexi | unclassified | 0 | 0 | **5.93*** | 0 |
|  | Cyanobacteria | unclassified | 0 | 1.87 | 1.3 | **0.52*** |
|  | Firmicutes | unclassified | **0.20**** | 0 | **0.05***** | **2.69***** |
|  | Gammaproteobacteria | unclassified | 0.62 | 0 | **0.15***** | 1.89 |
|  | Gemmatimonadetes | unclassified | 1.03 | 0 | 0 | **6.39*** |
|  | Planctomycetes | unclassified | 0 | 1.09 | **1.78*** | 0.57 |
|  | Deltaproteobacteria | Verrucomicrobia | 0 | **25.64*** | 0 | 0 |
|  | Gammaproteobacteria | Verrucomicrobia | **6.10*** | 0 | 0 | 0 |

**Table** **S3** Spearman correlations between the modules eigengenes of main modules (number of nodes > 5) in networks and environmental variables in two lake zones for bacterioplankton, microeukaryotes and inter-kingdom community.

| Bacterioplankton | Lake zones | NO. of modules | BM1 | BM2 | BM3 | BM4 | BM5 | BM6 | BM7 | BM8 | BM9 | BM10 | BM11 | BM12 | BM13 | BM14 | BM15 |
| --- | --- | --- | --- | --- | --- | --- | --- | --- | --- | --- | --- | --- | --- | --- | --- | --- | --- |
|  | MLB | TN | -0.26 | -0.04 | -0.24 | -0.31 | -0.3 | -0.15 | **0.76*** | 0.33 | 0.09 | 0.01 | **-0.82**** | -0.02 | **-0.66*** | 0.13 | -0.25 |
|  |  | TP | -0.27 | -0.09 | -0.32 | -0.28 | -0.24 | -0.24 | **0.79**** | 0.26 | 0.02 | 0.03 | **-0.79**** | 0.01 | **-0.67*** | 0.07 | -0.19 |
|  |  | NH_4_^+^-N | 0.21 | 0.31 | 0.37 | -0.07 | 0.3 | 0.45 | -0.01 | 0.61 | **0.67*** | 0.44 | 0.25 | 0.15 | 0.25 | 0.18 | 0.12 |
|  |  | NO_3_^-^-N | 0.38 | -0.59 | -0.07 | 0.27 | -0.07 | -0.18 | 0.18 | 0.27 | -0.2 | -0.25 | 0.12 | 0.12 | 0.14 | -0.31 | -0.02 |
|  |  | NO_2_^-^-N | 0.47 | 0.03 | 0.3 | -0.06 | 0.01 | 0.5 | -0.07 | **0.72*** | 0.31 | -0.02 | 0.19 | 0.15 | 0.13 | 0.14 | 0.10 |
|  |  | DOC | 0.01 | 0.59 | 0.21 | -0.19 | 0.02 | 0.49 | -0.35 | 0.12 | 0.07 | 0.09 | 0.14 | -0.01 | -0.08 | **0.64*** | 0.05 |
|  |  | pH | -0.53 | 0.04 | -0.05 | -0.13 | -0.18 | -0.37 | -0.28 | -0.54 | **-0.65*** | -0.44 | 0.03 | -0.45 | 0.03 | 0.05 | -0.26 |
|  | XKB | NO. of modules | BX1 | BX2 | BX3 | BX4 | BX5 | BX6 | BX7 | BX8 | BX9 | BX10 | BX11 |  |  |  |  |
|  |  | TN | **0.68*** | 0.43 | 0.2 | **0.72*** | 0.42 | **-0.76*** | **-0.73*** | 0.25 | 0.38 | **0.87**** | 0.55 |  |  |  |  |
|  |  | TP | **0.90***** | **0.76*** | -0.07 | **0.88***** | **0.65*** | **-0.64*** | -**0.89***** | 0.18 | 0.27 | **0.78**** | 0.52 |  |  |  |  |
|  |  | NH_4_^+^-N | -0.24 | -0.1 | 0.2 | 0.02 | -0.17 | -0.13 | 0.09 | 0.16 | 0 | 0.07 | 0.51 |  |  |  |  |
|  |  | NO_3_^-^-N | -0.33 | -0.05 | 0.59 | -0.08 | -0.24 | -0.26 | 0.08 | 0.61 | 0.26 | -0.25 | 0.38 |  |  |  |  |
|  |  | NO_2_^-^-N | -0.1 | 0.09 | 0.45 | 0.08 | -0.22 | -0.48 | 0.01 | 0.44 | 0.27 | 0.35 | 0.34 |  |  |  |  |
|  |  | DOC | -0.49 | -0.2 | -0.04 | -0.55 | -0.39 | 0.31 | 0.52 | 0.04 | -0.14 | -0.6 | -0.45 |  |  |  |  |
|  |  | pH | 0.61 | 0.32 | 0.22 | **0.67*** | 0.33 | -0.32 | **-0.71*** | -0.24 | 0.41 | 0.55 | 0.14 |  |  |  |  |
| Microeukaryotes | MLB | NO. of modules | BM1 | BM2 | BM3 | BM4 | BM5 |  |  |  |  |  |  |  |  |  |  |
|  |  | TN | 0.61 | -0.32 | -0.32 | 0.33 | 0.27 |  |  |  |  |  |  |  |  |  |  |
|  |  | TP | 0.62 | -0.35 | -0.28 | 0.27 | 0.2 |  |  |  |  |  |  |  |  |  |  |
|  |  | NH_4_^+^-N | -0.2 | **-0.64*** | 0.39 | -0.09 | -0.18 |  |  |  |  |  |  |  |  |  |  |
|  |  | NO_3_^-^-N | -0.16 | 0.04 | 0.18 | -0.28 | 0.18 |  |  |  |  |  |  |  |  |  |  |
|  |  | NO_2_^-^-N | -0.02 | -0.05 | 0.1 | 0.07 | 0.3 |  |  |  |  |  |  |  |  |  |  |
|  |  | DOC | 0.22 | 0.28 | -0.3 | 0.47 | 0.41 |  |  |  |  |  |  |  |  |  |  |
|  |  | pH | -0.01 | 0.1 | -0.24 | 0.05 | -0.11 |  |  |  |  |  |  |  |  |  |  |
|  | XKB | NO. of modules | MX1 | MX2 | MX3 | MX4 | MX5 | MX6 |  |  |  |  |  |  |  |  |  |
|  |  | TN | 0.41 | 0.43 | -0.35 | **-0.79*** | -0.41 | 0.10 |  |  |  |  |  |  |  |  |  |
|  |  | TP | 0.53 | 0.5 | -0.56 | **-0.94*** | -0.47 | 0.01 |  |  |  |  |  |  |  |  |  |
|  |  | NH_4_^+^-N | 0.21 | 0.1 | -0.01 | -0.07 | 0.35 | 0.04 |  |  |  |  |  |  |  |  |  |
|  |  | NO_3_^-^-N | 0.15 | 0.04 | **0.65*** | 0.25 | 0.33 | 0.31 |  |  |  |  |  |  |  |  |  |
|  |  | NO_2_^-^-N | 0.29 | 0.54 | 0.58 | 0.19 | 0.32 | 0.33 |  |  |  |  |  |  |  |  |  |
|  |  | DOC | -0.26 | -0.18 | 0.59 | **0.76*** | 0.27 | -0.02 |  |  |  |  |  |  |  |  |  |
|  |  | pH | 0.08 | 0.39 | -0.26 | -0.52 | -0.22 | 0.24 |  |  |  |  |  |  |  |  |  |
| Inter-kingdom | MLB | NO. of modules | 1 | 2 | 3 | 4 | 5 | 6 | 7 | 8 | 9 | 10 | 11 | 12 | 13 |  |  |
|  |  | TN | -0.20 | -0.03 | -0.27 | -0.24 | -0.30 | -0.32 | -0.26 | -0.02 | 0.30 | 0.05 | -0.25 | 0.02 | -0.02 |  |  |
|  |  | TP | -0.28 | -0.08 | -0.36 | -0.26 | -0.24 | -0.27 | -0.23 | 0.01 | 0.25 | -0.02 | -0.26 | -0.04 | -0.09 |  |  |
|  |  | NH_4_^+^-N | 0.40 | 0.07 | 0.28 | 0.18 | 0.30 | -0.12 | 0.43 | 0.15 | 0.04 | 0.28 | 0.32 | -0.09 | 0.51 |  |  |
|  |  | NO_3_^-^-N | -0.28 | **-0.64*** | -0.07 | 0.48 | -0.07 | -0.01 | 0.12 | 0.11 | -0.22 | -0.26 | 0.44 | -0.54 | -0.44 |  |  |
|  |  | NO_2_^-^-N | 0.41 | -0.05 | 0.23 | 0.48 | 0.01 | -0.27 | 0.11 | 0.15 | 0.19 | 0.25 | 0.43 | 0.10 | 0.19 |  |  |
|  |  | DOC | 0.6 | 0.67* | 0.22 | -0.09 | 0.02 | -0.13 | -0.20 | -0.01 | 0.52 | 0.36 | 0.03 | **0.72*** | 0.60 |  |  |
|  |  | pH | -0.32 | 0.07 | 0.04 | -0.48 | -0.18 | 0.02 | -0.28 | -0.45 | 0.01 | -0.20 | -0.59 | -0.07 | -0.18 |  |  |
|  | XKB | NO. of modules | 1 | 2 | 3 | 4 | 5 | 6 | 7 | 8 | 9 | 10 | 11 | 12 | 13 |  |  |
|  |  | TN | 0.56 | **0.72*** | 0.45 | 0.38 | 0.58 | -0.41 | **-0.70*** | 0.02 | **-0.81**** | **-0.72*** | -0.21 | 0.56 | -0.41 |  |  |
|  |  | TP | **0.81**** | **0.88***** | 0.62 | 0.32 | 0.61 | -0.61 | **-0.93***** | 0.04 | **-0.70*** | **-0.79**** | -0.22 | **0.79**** | -0.47 |  |  |
|  |  | NH_4_^+^-N | -0.23 | 0.02 | 0.09 | -0.05 | 0.10 | -0.10 | 0.09 | 0.18 | -0.17 | 0.20 | -0.22 | -0.32 | 0.35 |  |  |
|  |  | NO_3_^-^-N | -0.42 | -0.08 | 0.12 | 0.27 | 0.08 | **0.70*** | 0.25 | -0.19 | -0.20 | 0.31 | 0.47 | -0.07 | 0.33 |  |  |
|  |  | NO_2_^-^-N | -0.18 | 0.08 | 0.32 | 0.18 | 0.44 | 0.59 | 0.12 | -0.23 | -0.43 | -0.23 | 0.62 | 0.10 | 0.42 |  |  |
|  |  | DOC | -0.44 | -0.55 | -0.21 | -0.20 | -0.31 | **0.71*** | 0.62 | -0.33 | 0.39 | 0.37 | 0.52 | -0.22 | 0.27 |  |  |
|  |  | pH | 0.45 | **0.67*** | 0.19 | 0.58 | 0.43 | -0.30 | **-0.81**** | 0.41 | -0.31 | -0.56 | 0.12 | 0.59 | -0.22 |  |  |

*** *P*< 0.001; ** *P*< 0.01; * *P*< 0.05

TN, total nitrogen; TP, total phosphorus; NH_4_^+^-N, ammonia nitrogen; NO_3_^-^-N, nitrate nitrogen; NO_2_^-^-N, nitrite; DOC, dissolved organic carbon.

**Table S4 The longitude and latitude of the sampling sites in Meiliang Bay (MLB) and Xukou Bay (XKB) in Lake Taihu, China.**

| Sampling sites | longitude and latitude | Sampling sites | longitude and latitude |
| --- | --- | --- | --- |
| MLB1 | 31.50723N 120.15620E | XKB1 | 31.21658N 120.43617E |
| MLB2 | 31.50730N 120.17654E | XKB2 | 31.00792N 120.42356E |
| MLB3 | 31.50686N 120.19826E | XKB3 | 31.16039N 120.40131E |
| MLB4 | 31.48094N 120.15508E | XKB4 | 31.18281N 120.42764E |
| MLB5 | 31.48106N 120.17658E | XKB5 | 31.16473N 120.36626E |
| MLB6 | 31.48067N 120.19810E | XKB6 | 31.16700N 120.40433E |
| MLB7 | 31.45550N 120.15643E | XKB7 | 31.14360N 120.37764E |
| MLB8 | 31.41680N 120.21758E | XKB8 | 31.16810N 120.36927E |
| MLB9 | 31.42995N 120.15661E | XKB9 | 31.15329N 120.37500E |
| MLB10 | 31.42054N 120.21655E | XKB10 | 31.11450N 120.40291E |

R code 1:

calculating O/R

nodes=read.table("network_nodes.txt",header=T)

edges=read.table("network_graph.sif",header=F)

phylum=levels(nodes$phylum)

Nphy=array(NA,length(phylum))

for (i in 1:length(phylum)){

Nphy[i]=sum(nodes$phylum==phylum[i])}

Nephyij=matrix(NA,length(phylum),length(phylum))

for(jj in 1:(length(phylum))){

for(ii in 1:length(phylum)){

Tphyi=as.character(nodes$OTU[nodes$phylum==phylum[ii]])

Tphyj=as.character(nodes$OTU[nodes$phylum==phylum[jj]])

Nephyij[ii,jj]=sum(edges[,1]%in%Tphyi&edges[,3]%in%Tphyj)}}

Nephyij=Nephyij+t(Nephyij)-Nephyij*diag(length(phylum))

SX=apply(Nephyij,1,sum)>0

Lab1=matrix(rep(phylum,times=length(phylum)),length(phylum),length(phylum))

Lab2=matrix(rep(phylum,each=length(phylum)),length(phylum),length(phylum))

N1=matrix(rep(Nphy,times=length(phylum)),length(phylum),length(phylum))

N2=matrix(rep(Nphy,each=length(phylum)),length(phylum),length(phylum))

Nephyij=Nephyij[SX,SX]

Lab1=Lab1[SX,SX]

Lab2=Lab2[SX,SX]

N1=N1[SX,SX]

N2=N2[SX,SX]

Nephyii=diag(Nephyij)

Cbii1=cbind(phylum[SX],phylum[SX],Nphy[SX],Nphy[SX],Nephyii,Oberved=Nephyii/dim(edges)[1],Randtheo=(Nphy[SX]-1)*(Nphy[SX])/(dim(nodes)[1]*(dim(nodes)[1]-1)))

Lab1ij=array(NA,(length(Nephyii)-1)*length(Nephyii)/2)

Lab2ij=array(NA,(length(Nephyii)-1)*length(Nephyii)/2)

N1ij=array(0,(length(Nephyii)-1)*length(Nephyii)/2)

N2ij=array(0,(length(Nephyii)-1)*length(Nephyii)/2)

NephyPij=array(NA,(length(Nephyii)-1)*length(Nephyii)/2)

for(m in 1:(length(Nephyii)-1)){

for(n in (m+1):length(Nephyii)){

Lab1ij[(n-2)*(n-1)/2+m]=Lab1[m,n]

Lab2ij[(n-2)*(n-1)/2+m]=Lab2[m,n]

N1ij[(n-2)*(n-1)/2+m]=N1[m,n]

N2ij[(n-2)*(n-1)/2+m]=N2[m,n]

NephyPij[(n-2)*(n-1)/2+m]=Nephyij[m,n] }}

Cbij1=cbind(Lab1ij,Lab2ij,N1ij,N2ij,NephyPij,Oberved=NephyPij/dim(edges)[1],Randtheo=N1ij*N2ij*2/(dim(nodes)[1]*(dim(nodes)[1]-1)))

write.csv(Cbii1,"p_S4ii.csv")

write.csv(Cbij1,"p_S4ij.csv")

###################################################

library(igraph)

nodes=read.table("network_nodes.txt",header=T)

edges=read.table("network_graph.sif",header=F)

Nn=dim(nodes)[1]

Ne=dim(edges)[1]

phylum=levels(nodes$phylum)

Lphy=length(phylum)

nodes$OTU=1:Nn

N=1000

CS=matrix(0,Lphy,Lphy)

CN=matrix(0,Lphy,Lphy)

for(i in 1:N){

g1=erdos.renyi.game(Nn,Ne,'gnm',weight=T,mode="undirected")

g=simplify(g1)

edges[,1]=get.edgelist(g)[,1]

edges[,3]=get.edgelist(g)[,2]

Nephyij2=matrix(NA,Lphy,Lphy)

for(jj in 1:(Lphy)){

for(ii in 1:Lphy){

Tphyi=nodes$OTU[nodes$phylum==phylum[ii]]

Tphyj=nodes$OTU[nodes$phylum==phylum[jj]]

Nephyij2[ii,jj]=sum(edges[,1]%in%Tphyi&edges[,3]%in%Tphyj)}}

Nephyij2=Nephyij2+t(Nephyij2)-Nephyij2*diag(Lphy)

tamp=Nephyij2>=Nephyij

CN=CN+tamp

CS=CS+Nephyij2

}

ECS=CS/N

PCN=CN/N

Nephyij2=ECS

Lab1=matrix(rep(phylum,times=Lphy),Lphy,Lphy)

Lab2=matrix(rep(phylum,each=Lphy),Lphy,Lphy)

N1=matrix(rep(Nphy,times=Lphy),Lphy,Lphy)

N2=matrix(rep(Nphy,each=Lphy),Lphy,Lphy)

Nephyii=diag(Nephyij2)

CNphyii=diag(PCN)

Cbii=cbind(phylum,phylum,RandomER=Nephyii/dim(edges)[1])

Lab1ij=array(NA,(length(Nephyii)-1)*length(Nephyii)/2)

Lab2ij=array(NA,(length(Nephyii)-1)*length(Nephyii)/2)

N1ij=array(0,(length(Nephyii)-1)*length(Nephyii)/2)

N2ij=array(0,(length(Nephyii)-1)*length(Nephyii)/2)

NephyPij=array(NA,(length(Nephyii)-1)*length(Nephyii)/2)

CNphyPij=array(NA,(length(Nephyii)-1)*length(Nephyii)/2)

for(m in 1:(length(Nephyii)-1)){

for(n in (m+1):length(Nephyii)){

Lab1ij[(n-2)*(n-1)/2+m]=Lab1[m,n]

Lab2ij[(n-2)*(n-1)/2+m]=Lab2[m,n]

N1ij[(n-2)*(n-1)/2+m]=N1[m,n]

N2ij[(n-2)*(n-1)/2+m]=N2[m,n]

NephyPij[(n-2)*(n-1)/2+m]=Nephyij2[m,n]

CNphyPij[(n-2)*(n-1)/2+m]=PCN[m,n]}}

Cbij=cbind(Lab1ij,Lab2ij,RandomER=NephyPij/dim(edges)[1])

S4ii=read.table("p_S4ii.csv",header=T,row.names=1,sep=",")

S4ij=read.table("p_S4ij.csv",header=T,row.names=1,sep=",")

RdEii=S4ii$Randtheo

Rpii=S4ii$Randtheo

for(i in 1:dim(S4ii)[1]){

for(j in 1:dim(Cbii)[1]){

if(S4ii[i,1]==Cbii[j,1]&S4ii[i,2]==Cbii[j,2]){RdEii[i]=Cbii[j,3]

Rpii[i]=CNphyii[j]}

}}

RdEij=S4ij$Randtheo

Rpij=S4ij$Randtheo

for(i in 1:dim(S4ij)[1]){

for(j in 1:dim(Cbij)[1]){

if(S4ij[i,1]==Cbij[j,1]&S4ij[i,2]==Cbij[j,2]){RdEij[i]=Cbij[j,3]

Rpij[i]=CNphyPij[j]}

}}

ORii=S4ii[,6]/as.single(RdEii)

ORij=S4ij[,6]/as.single(RdEij)

Rpii[Rpii>0.5]=1-Rpii[Rpii>0.5]

Rpij[Rpij>0.5]=1-Rpij[Rpij>0.5]

CII=cbind(Node1lab=as.character(S4ii[,1]),Node2lab=as.character(S4ii[,2]),Node1=as.character(S4ii[,3]),Node2=as.character(S4ii[,4]),Num_Observed_edge=S4ii[,5],Observed=S4ii[,6],RandomER=RdEii,RandomTheo=S4ii[,7],P=Rpii,OR=ORii)

CIJ=cbind(Node1lab=as.character(S4ij[,1]),Node2lab=as.character(S4ij[,2]),Node1=as.character(S4ij[,3]),Node2=as.character(S4ij[,4]),Num_Observed_edge=S4ij[,5],Observed=S4ij[,6],RandomER=RdEij,RandomTheo=S4ij[,7],P=Rpij,OR=ORij)

S4=rbind(CII,"",CIJ)

write.csv(S4,"O/R.csv")

R code 2:

calculating Pi, Zi

MR=read.table('MR.txt',header=T,row.names=1)

tMR1=t(MR)

library(Hmisc)

library(qvalue)

X1=rcorr(tMR1,type="spearman")

XR1=X1$r

XN1=X1$n

XP1=X1$P

XP11=matrix(XP1,nrow=dim(XR1)[1],ncol=dim(XR1)[1])

XQ11=qvalue(XP11)$qvalues

allX=XR1;allX[,]=0

for(i in 1:dim(XR1)[1]){

for(j in 1:dim(XR1)[2]){

if(abs(XR1[i,j])>0.9&abs(XR1[i,j])<1&i!=j&XP11[i,j]<0.01&XQ11[i,j]<0.05){allX[i,j]=XR1[i,j]}

}}

allX1=allX[apply(abs(allX),1,sum)!=0,apply(abs(allX),2,sum)!=0]

write.csv(allX1,"network_R.csv",quote=F)

G1=graph.adjacency(network_R,mode="undirected",weighted=TRUE)

seqdeg=degree(G1)

Nnodes=length(seqdeg)

Z=seqdeg

Z[]=0

P=Z

Wtc=fastgreedy.community(G1)

Membership=membership(Wtc)

Seq=seq(1:Nnodes)

for(i in 1:Nnodes){

L=Membership==Membership[i]

neighbs=neighbors(G1,i)

Kis=sum(L[neighbs])

SUM=0

SUMsq=0

SUMP=0

Miv=Seq[L]

for(j in 1:sum(L)){

neighbsj=neighbors(G1,Miv[j])

Kjs=sum(L[neighbsj])

SUM=SUM+Kjs

SUMsq=SUMsq+Kjs^2

}

Z[i]=(Kis-SUM/sum(L))/sqrt(SUMsq/sum(L)-(SUM/sum(L))^2)

if(Kis-SUM/sum(L)==0){Z[i]=0}

for(k in 1:max(Membership)){

Lp=Membership==k

Kisp=sum(Lp[neighbs])

SUMP=SUMP+(Kisp/seqdeg[i])^2}

P[i]=1-SUMP

}

attribute_node=cbind(degree=seqdeg,module=Membership,Pi=P,Zi=Z)

write.csv(attribute_node,"attribute_node.csv",quote=F)
